# Translation and validity of the Multidimensional Individual and Interpersonal Resilience Measure

**DOI:** 10.1590/0034-7167-2022-0696

**Published:** 2023-10-09

**Authors:** Jéssica Diniz Rodrigues Ferreira, Mariana Figueiredo Miranda, Millena Figueiredo Miranda, Marco Aurélio Romano-Silva, Maria Aparecida Camargos Bicalho, Bernardo de Mattos Viana

**Affiliations:** I Universidade Federal de Minas Gerais. Belo Horizonte, Minas Gerais, Brazil; II Centro de Tecnologia em Medicina Molecular (CTMM) e INCT - NeuroTecR. Belo Horizonte, Minas Gerais, Brazil; III Universidade Federal de Minas Gerais, Hospital das Clínicas. Belo Horizonte, Minas Gerais, Brazil

**Keywords:** Resilience, Psychological, Validation Study, Aged, Translations, Psychometrics., Resiliência Psicológica, Estudo de Validação, Idoso, Tradução, Psicometria., Resiliencia Psicológica, Estudio de Validación, Anciano, Traducción, Psicometría.

## Abstract

**Objective:**

to translate, culturally adapt and validate the Multidimensional Individual and Interpersonal Resilience Measure to Brazilian Portuguese.

**Method:**

after initial translation, the pre-final version underwent rigorous cultural adaptation procedures. As a result, the final adapted version was submitted to a validity study.

**Results:**

adaptation procedures provided equivalence between the pre-final and the original versions in semantic, idiomatic, experiential and conceptual terms. A total of 187 older adults were included in the validity study. Exploratory factorial analysis (EFA) generated a model of five factors ((RMSEA = 0.030; TLI = 0.959; X^2^ = 151.590 p> 0.05). Final version showed adequate consistency (Cronbach’s α = 0.705) and test-retest reliability (ICC=0.835). No statistically significant correlation was found between resilience and sociodemographic and epidemiological variables assessed in this study.

**Conclusion:**

EMRII-BR is a valid and reliable instrument for measuring resilience in Brazilian older adults.

## RESUMO

**Objetivo:**

traduzir, adaptar culturalmente e validar o Multidimensional Individual and Interpersonal Resilience Measure para o português brasileiro.

**Método:**

após a tradução inicial, a versão pré-final passou por rigorosos procedimentos de adaptação cultural. Como resultado, a versão final adaptada foi submetida a um estudo de validade.

**Resultados:**

os procedimentos de adaptação proporcionaram equivalência entre as versões pré-final e original em termos semânticos, idiomáticos, experienciais e conceituais. Um total de 187 idosos foram incluídos no estudo de validade. A análise fatorial exploratória (AFE) gerou um modelo de cinco fatores ((RMSEA = 0,030; TLI = 0,959; X^2^ = 151,590 p> 0,05). A versão final apresentou consistência adequada (α de Cronbach = 0,705) e confiabilidade teste-reteste (ICC=0,835). Não foi encontrada correlação estatisticamente significativa entre a resiliência e as variáveis sociodemográficas e epidemiológicas avaliadas neste estudo.

**Conclusão:**

o EMRII-BR é um instrumento válido e confiável para mensurar a resiliência em idosos brasileiros.

## RESUMEN

**Objetivo:**

traducir, adaptar culturalmente y validar el Multidimensional Individual and Interpersonal Resilience Measure para el portugués brasileño.

**Método:**

después de la traducción inicial, la versión pre-final pasó por rigurosos procedimientos de adaptación cultural. Como resultado, la versión final adaptada fue sometida a un estudio de validez.

**Resultados:**

los procedimientos de adaptación proporcionaron equivalencia entre las versiones pre-final y original en términos semánticos, idiomáticos, experienciales y conceptuales. Un total de 187 ancianos fueron incluidos en el estudio de validez. El análisis factorial exploratorio (AFE) generó un modelo de cinco factores ((RMSEA = 0,030; TLI = 0,959; X^2^ = 151,590 p> 0,05). La versión final mostró adecuada consistencia (α de Cronbach = 0,705) y confiabilidad prueba-reprueba (ICC=0,835). No se encontró correlación estadísticamente significativa entre la resiliencia y las variables sociodemográficas y epidemiológicas evaluadas en este estudio.

**Conclusión:**

EMRII-BR es un instrumento válido y confiable para medir la resiliencia en ancianos brasileños.

## INTRODUCTION

The world’s population is aging, primarily due to the decline in fertility rates, associated with increased life expectancy^(1-3)^. In Brazil, as estimated by the Brazilian Institute of Geography and Statistics (IBGE - *Instituto Brasileiro de Geografia e Estatística*), the proportion of individuals over 65 years is expected to be 13.54% by 2030, whilst this measure was estimated in 10.49% in 2022^(4)^. The country is rapidly going towards an older demographic profile, marked by many challenges^(5)^, including the increase in chronic noncommunicable diseases, leading to social and financial burdens^(6)^. On the other hand, aging may be considered a plastic process. Current models suggest a dialectic relation between the amount and/or strength of biopsychosocial resources and vulnerabilities during the life cycle, which may be moderated by protective factors, such as resilience^(7)^.

Resilience is the individual and interpersonal ability to overcome, adapt and develop personal knowledge in face of adversities. It is a multidimensional and complex construct related to genetic, biological, psychological and environmental factors^(8-10)^. The main protective factors related to individual resilience are realistic optimism, ability to face fears, moral direction, religion and spirituality, reference models of conduct, physical activity, cognitive and emotional flexibilities, perception of meaning and purpose for lived experiences and self-efficacy^(8,11)^. However, older adults often experience a series of non-normative and age-related changes, such as decline in health and functionality. Such adversities range from everyday challenges to highly stressful experiences that may impair older adults and their entire family and its social system. The concept of family resilience is based on observations that the family as a system adapts, adjusts, recovers and strengthens in the face of challenging situations. From this point of view, more consistent with the construct’s multidimensional nature, concept understanding now includes multigenerational factors. Resilience factors come to be understood as processes, including also family communication, belief systems, spirituality, flexibility, family agreement, routines and social support^(8)^.

The combination of factors such as genetic predisposition, socio-environment, family history, early traumatic events and chronic illness and their treatment increases the vulnerability for mental disorders, including anxiety and depressive disorders^(12)^. Despite these combination factors, individuals perceive and cope with chronic stress differently, depending on individual abilities to adapt. In this context, resilience levels differ between older people and are associated with more favorable characteristics. For instance, resilience has been associated with involvement in advanced activities of daily living, absence/fewer depressive and anxious symptoms, less self-reported illnesses, higher quality of life, and lower use of health care and health expenses^(13-17)^.

During the COVID-19 pandemic, concerns about older adults’ mental health have been frequent. Despite that, it has been shown that older adults had higher resilience than emerging adults, which was positively associated with mental health^(18)^. In this population, resilience has also been demonstrated to predict lower anxiety levels during the pandemic as well as high resilience was associated with a lower perception of threat by COVID-19^(19-20)^.

In this context, the theme of the 2022 International Day of Older People is “Resilience of Older Persons in a Changing World”^(21)^. This exemplifies the increasing need to discuss this subject. There is also a growing interest for multicentric and multicultural research projects focusing on resilience. This implies to a growing demand for assessment instruments applicable to different cultures and/or languages, preferably adapted and validated from the original version^(22)^. In this regard, it is valid to emphasize the scarcity of instruments to measure both individual and interpersonal dimensions of resilience and that are specifically validated for Brazilian older adults.

## OBJECTIVE

To validate the translated and culturally adapted version of the Multidimensional Individual and Interpersonal Resilience Measure (MIIRM).

## METHODS

### Ethical aspects

This study was conducted in accordance with national and international ethics guidelines and approved by the Research Ethics Committee of the *Universidade Federal de Minas Gerais*, whose letter of approval is attached to this submission. The Informed Consent Form was obtained from all individuals involved in the study through written procedure.^(1)^

### Study design, time period and place

The present study had a cross-sectional design and used the STrengthening the Reporting of OBservational studies in Epidemiology (STROBE) statement to guide research report. It was conducted in Belo Horizonte, Minas Gerais, Brazil, from October 2017 to December 2018.^(1)^

### Population or sample; inclusion and exclusion criteria

The sample was composed by individuals 60 years old and over with different levels of education, living in the metropolitan area of Belo Horizonte, with capacity to inform consent and to answer the questionnaire. Older adults with diagnosis of dementia or a terminal illness, residents in a long-term care institution, and/or with need for frequent hospitalization were excluded.^(1)^

### Study protocol

The process of translation, cultural adaptation and validity followed the guidelines proposed by Beaton *et al*. (2000) and Gorenstein *et al*. (2016)^(22-23)^. Initial translation was performed by two independent translators, fluent in English and Brazilian Portuguese, one of them being a specialist in the area of the construct. Two initial versions in Brazilian Portuguese were obtained (T1 and T2). After a meeting between the translators and the main investigators, final considerations were discussed to achieve the synthesis version (T1.2). Thereafter, version T1.2 was submitted to back-translation, conducted by two independent translators, in order to verify semantic equivalence between the translated versions and the original instrument. As a result, two independent back-translations were produced (R1 and R2).

All five versions produced, so as the original MIIRM were submitted to assessment by an expert committee, composed by eight specialists of different areas: one occupational therapist; two psychogerontologists; three psychogeriatrics; one physical educator; and one geriatrician. After careful discussion, participants agreed on the following aspects: semantic equivalence; idiomatic equivalence; experimental equivalence; and conceptual equivalence.

The pre-final version of the MIIRM, obtained after expert committee conclusion, was then submitted to a pilot study, the last phase before the validity study. A total of 26 older adults recruited from the community were interviewed. As a result of this process, after final adjustments, we established the final version of *Escala Multidimensional de Resiliência Individual e Interpessoal - Brasil* (EMRII-BR).

The validity study was conducted between March and December 2018, to confirm if the EMRII-BR had adequate psychometric features for use with the target population. Convenience sampling was applied in the Clinical Hospital of the *Universidade Federal de Minas Gerais*. In this phase, the translated and culturally adapted instrument was part of an assessment protocol, which included sociodemographic information, self-report of health conditions, and the Mini-Mental State Examination (MMSE). The study protocol was applied by a psychologist and a team of trained collaborators.^(1)^

### Results analysis, and statistics

Description statistics were used for continuous and categorical variables. Exploratory factor analysis (EFA) was selected for the present data, considering that the scale is relatively recent in the literature, since the original study was the only one to investigate its psychometric properties. Therefore, once EMRII-BR underwent modifications through translation and adaptation processes, EFA could achieve a new factorial model.^(1)^

Most EMRII-BR items have positive scores, not reversed. For the analysis, the five items of reversed scoring were duly transformed in the database, for instance: item 6, when receiving a score 2 (1-5 Likert-type scale) by one given individual, was replaced by score 4 ^(1)^

Descriptive analysis, correlation and reliability tests were made in the SPSS, while EFA was made through JASP software.^(1)^

## RESULTS

The process of cultural adaptation resulted in a translated version. Through a comprehensive review by an expert committee, the pre-final version of EMRII-BR acquired some detailed improvements, essential to experiential equivalence, such as emphasis on expressions and words that would be better understood by older adults of different educational levels. Also, conceptual equivalence analysis raised discussions related to item 12. This item relates higher financial, educational and social positions with higher resilience scores. Most of the committee experts hypothesized that many Brazilian older adults are not necessarily influenced by those factors in subjective resilience perception. At last, it was suggested that during assessment the interviewer explained, if necessary, the difference between religiosity and spirituality, since it may not be completely understood by all individuals. ^(1)^

After consensus, the pre-final version was then submitted to a pilot-study, which consisted of assessment of 26 older adults. In the validity study, 187 outpatients were included. Both sample characteristics are described in Table 1. ^(1)^

**Table 1:** Sample characteristics, pilot-study and validity study

	PILOT STUDY (N=26)		VALIDITY STUDY (N=187)	
	**Mean** **(SD)/n (%)**	**Min.-Max.**	**Mean** **(SD)/n (%)**	**Min.-Max.**
Age	73.35 (1,374)	62-88	69,23 (6,912)	60 - 91
Sex				
Male	17 (65.4%)		65 (34.8)	
Female	9 (34.6%)		122 (65.2)	
Years of education	8.15 (1,003)	0-17	7.16 (4,996)	0 - 25
Marital status				
Married			99 (52.9)	
Widower/widow			45 (24.1)	
Single			22 (11.8)	
Divorced			21 (11.2)	
Occupational status				
Retired			138 (73.8)	
Current job			57 (30.5)	
Religion				
Yes			180 (96.3)	
No			7 (3.7%)	
EMRII-BR (total score)			90.38 (11,067)	59 - 117

EMRII-BR - Escala Multidimensional de Resiliência Individual e Interpessoal.

The sample was considered adequate for factor analysis, according to Kaiser-Meyer-Olkin (KMO) test results greater than 0.5 (0.661) and Bartlett’s sphericity test, with a significance value less than 0.001^(1,24)^.

Parallel analysis suggested a 5-factor model, while the results on the screen plot suggested a 3-factor model. Kaiser criteria suggests the extraction of a single factor for eigenvalues above 1. Since the original study found 8 factors for MIIRM, parallel analysis results are the closest.^(1)^

In order to achieve a simpler structure, according to parallel analysis, items 3 (“I usually recover quickly after illness or other life difficulties”), 5 (“Before criticizing someone, I try to put myself in her place and imagine how she would feel”), 7 (“I do not comfort people when they need it”), 8 (“When people are talking to me, I find myself wishing they would leave”) and 14 (“How often do you feel lonely?”) were excluded from the adapted version. For all those items, component weight was not greater than 0.3 (r = 0.3 corresponds to an average effect size; approximately 10% of the item’s variance is explained by the corresponding factor). Each item is expected to present a weight for a single factor. The item that presented adequate weight for more than one factor or for no factor was removed from the analysis. In this case, items excluded as described above presented weight for none of the factors. Table 2 shows detailed weight components.^(1)^

**Table 2:** Components weights

Item	RC 1	RC 2	RC 3	RC 4	RC 5	Uniqueness
EMRII_BR_1	0.127	-0.058	0.499	-0.167	0.192	0.725
EMRII_BR_10	0.027	-0.018	-0.108	0.715	0.068	0.505
EMRII_BR_11	0.038	0.158	0.036	0.466	0.006	0.721
EMRII_BR_12	0.027	0.129	-0.080	0.095	0.413	0.770
EMRII_BR_13	-0.034	-0.038	0.293	-0.064	0.783	0.349
EMRII_BR_15	0.348	-0.216	0.105	0.206	-0.122	0.745
EMRII_BR_16	0.507	-0.109	-0.024	0.025	0.005	0.746
EMRII_BR_17	0.574	0.175	-0.092	0.091	0.115	0.515
EMRII_BR_18	0.785	0.086	0.024	-0.001	-0.021	0.352
EMRII_BR_19	-0.211	0.973	-0.039	0.164	0.072	0.076
EMRII_BR_2	-0.053	-0.011	0.320	0.056	0.069	0.883
EMRII_BR_20	0.215	0.351	0.032	-0.046	0.010	0.798
EMRII_BR_21	0.139	0.111	0.385	-0.034	0.031	0.788
EMRII_BR_22	0.017	-0.030	0.532	0.000	-0.140	0.699
EMRII_BR_4	-0.178	-0.088	0.384	0.195	0.100	0.768
EMRII_BR_6	-0.098	0.177	0.351	-0.005	-0.269	0.769
EMRII_BR_9	0.092	0.021	0.126	0.510	-0.028	0.628

* JASP software; EMRII-BR - Escala Multidimensional de Resiliência Individual e Interpessoal.

Both model fit and residual statistics were excellent, with Tucker-Lewis Index (TLI) greater than 0.95 (TLI 0.959) and Root Mean Square Error less than 0.06 (RMSEA 0.03). Moreover, chi-square value (X^2^) is the most traditional measure to assess general model fit. A good model fit is indicated by a statistically non-significant value (p> 0.05). In the present study, therefore, X^2^ value suggested a good model fit (p=0.105).^(1)^

Regarding reliability, the five-factor model showed an acceptable Cronbach’s alpha (α = 0.705). Analyzed separately, individual factors did not present satisfactory reliability (α <0.7). ^(1)^

EMRII-BR total scores followed a normal distribution both in test and retest, according to Shapiro-Wilk test (p> 0.05), therefore meeting criteria for intraclass correlation coefficient analysis (ICC). Results showed good reliability (ICC> 0.75 and <0.90). Also, regarding inter-rater reliability, results for each pair of evaluators indicated a practically absolute reliability. ICC and 95% confidence intervals analysis were based on mixed two factors and absolute agreement models.^(1)^

## DISCUSSION

The process of cultural adaptation resulted in a new and adequate version for Brazilian older adults. Translation, cross-cultural adaptation and validity methodologies must aim at an optimized instrument for the target population, at the same time respecting equivalence principles when compared to the original version. The present study followed the Beaton *et al.* (2000) and Gorenstein *et al.* (2016)^(22-23)^ recommendations.

Translating procedures resulted in little disagreement between translators for the synthetic version, since the original scale presented few idiomatic expressions and conceptual differences from Brazilian Portuguese. Original scoring was widely discussed by the expert committee. After consensus, a 5-point Likert scale was maintained for items 1 to 11 and 14 to 20; items 12 and 13 scored from 1 to 10; and items 21 and 22 were maintained as a 4-point Likert scale, as proposed in the original version.

There is still little consensus in literature on cross-cultural adaptation methodology in terms of which sequence or selection of steps can be considered more effective. Therefore, it is recommended to use several techniques, depending on feasibility, sample characteristics and psychometric properties^(23,25-26)^. Furthermore, cross-cultural research presents specific methodological challenges that, for the most part, refer to quality of translation procedures and comparability in different cultural and ethnic groups. Translation and cultural adaptation are independent processes, but both equally are correlated to the final version quality, and then must be strictly planned and executed in order to achieve maximum equivalence in all its aspects^(27)^.

In addition, there was a focus on adapting the language to the target population in terms of education and current expressions most used by older adults. According to Gorenstein *et al*. (2016)^(23)^, the main challenge in cross-cultural research is to develop a methodology that integrates both global perspective and cultural validity. The expert committee also raised questions about the reverse scoring items. Theoretically, items scored using a Likert scale are generally ranked from “lowest” to “highest”, with the positive pole on the right and the negative pole on the left. When this ordering is reversed, items are considered to have a reverse score, i.e., respondents need to invert their thinking when choosing an answer option. Some authors show that when English speakers attribute items to reverse scores, there is a slight tendency towards higher scores, suggesting a bias in this type of question^(28-30)^. In our study, some participants indicated less understanding of these items during assessment, which was either reported by the participants themselves or observed by the interviewers, both in pilot and validity studies. These items seem to have a negative effect on assessment by interrupting the line of reasoning when individuals seem to have already understood the pattern of questions and answers. Consequently, there is also an effect on the interviewer, since these items may cause concern and a slight tendency to explain the question, in addition to standard description. Finally, from a statistical point of view, items of reverse scoring seem to contribute to a less simple factorial model. In our sample, a total of three from five items of reverse scoring were excluded in factorial analysis so as to achieve a better structure.

Thus, conceptual equivalence analysis raised discussions related to item 12, which suggests that higher financial, educational and social positions might have positive correlations with higher resilience scores. Most of the expert committee hypothesized that many Brazilian older adults would not be necessarily influenced by those factors in subjective resilience perception.

In the original study of the scale, data collection was performed by mail, while in the present study it was carried out through face-to-face interviews. We have opted for this methodology aiming to include illiterate individuals and those with low educational level. Interviews were conducted with 195 older people, of which 8 protocols were excluded from the analyzes, as they did not meet all inclusion criteria. One of the most recent recommendations for sample size in validity studies is 10 times the number of items on the scale^(31)^. EMRII-BR, during the validity study, presented 22 items, so it was intended to reach a sample of 220 respondents. Although the initial goal has not been reached, it was considered sufficient for data analysis, as it is higher than previous recommendations by Hair *et al*. (2006)^(24)^ (5 times the number of items on the scale).

Although most of the correlations presented are not statistically significant, some trends have drawn attention, for instance: positive correlation between total EMRII-BR score and age (p = 0.658; rho = 0.033); and negative correlation between total EMRII-BR score and education level (p = 0.134; rho = -0.110) and total Mini-Mental score (p = 0.422; rho = -0.059).

Mean age of the older adults included in the present study was 69.23 years (SD = 6.912), which is considered relatively low. In the original study, participants’ mean age was 77.35 years (SD = 12.2 years), with 48.6% of the sample being female, and the majority having a high level of education^(8)^. The predominance of “young older adults” in the sample may influence higher levels of resilience, moderated by a more preserved functionality, when compared to oldest-old individuals. It would be interesting for further studies to compare resilience levels in older adults, divided by age into life-stages groups, such as youngest-old, middle-old and oldest-old.

EFA was carried out. The fit index of the sample in the present study was adequate. The KMO test value was 0.661. The original study found 0.68, with results greater than 0.5 being adequate^(24)^. Bartlett’s sphericity test showed statistical significance <0.001 as well as that of the original study (p <0.01)^(8)^. With regard to model fit, there are several fit index currently available in literature, which may contribute to relatively lack of reference^(32)^. To assess the quality of the model presented in this study, TLI greater than 0.95, RMSEA less than 0.06 and chi-square test with a statistically non-significant value (p> 0.05) were used.

Oblique rotation, promax subtype, was used in order to simplify and clarify data structure. There are several types of rotation methods, organized into two major classifications: oblique and orthogonal. Oblique rotation methods allow factors to correlate, while orthogonal rotation methods assume that there is no relationship between the factors. In social sciences, it is generally expected that there is a correlation between factors; therefore, oblique rotation is often preferred in this area of study as it offers a more accurate solution. Furthermore, if there is no relationship between the factors, oblique rotation will offer results very similar to those obtained through orthogonal rotation^(33)^. In the MIIRM original study, orthogonal rotation, varimax subtype was used, since intercorrelations between the eight factors found through oblique rotation were low^(8)^. Nevertheless, a different approach was chosen, as it is believed that once grouped on the same scale, it can be assumed that the latent constructs are correlated.

The factorial structure found in our study included a total of five factors: (1) access to support network; (2) interpersonal relationship; (3) self-efficacy; (4) optimism; (5) perceived social and economic resources. This structure is simpler when compared to the original study, in which eight factors were obtained. Originally, factors 2 and 5 grouped only two items each, which makes these factors weak or unstable, according to some authors^(33)^.

Regarding internal consistency, the five-factor model presented an acceptable Cronbach’s alpha (α = 0.705), similar to the value found in the original study (α = 0.720)^(8)^. Although the α value obtained was relatively low, the factorial structure presented is simpler and more adequate. To reach the final structure, five items were removed, totaling 17 items, grouped into five factors. Nevertheless, these changes did not appear to significantly affect the latent constructs initially proposed. Thus, when naming the five factors found, it was decided to keep the corresponding denominations suggested in the original study, due to theoretical similarities identified.

Interrater and test-retest reliability were calculated using ICC. Total EMRII-BR scores obtained for each evaluator (equivalence between evaluators) and at different times (test-retest) were compared. Inter-rater reliability aims to verify equivalence in scale scores by different observers, i.e., once applied by different raters, the scores obtained are significantly in agreement. Estimates for ICC and 95% confidence intervals were calculated, based on a mixed model of two factors and absolute agreement. Values found for each of the three pairs of evaluators indicated a practically absolute reliability or very close to 1. The method used in this study to measure inter-rater reliability allowed only to verify equivalence between scores. Scoring biases were not assessed, which, however, tend to be minimized by training and also by the fact that scoring is based on reading the questions, and not on the interviewer’s interpretation. Test-retest reliability, on the other hand, aimed to verify data temporal stability, such as how much the results remain consistent after a certain period of time. Total EMRII-BR scores, both in test and retest, followed a normal distribution, according to Shapiro-Wilk test (p> 0.05). Therefore, they met criteria for calculating ICC, which showed good reliability (ICC> 0.75 and <0.90).

Some of the most used indicators to verify reliability include Cohen’s Kappa, Fleiss’s Kappa and ICC. Cohen’s Kappa and Fleiss’s Kappa are indicated for cases in which there are only two evaluators or only one evaluator, respectively. For both, variables analyzed must be categorical. ICC, on the other hand, is indicated for contexts in which there are more than two evaluators and variables analyzed are continuous. Therefore, this indicator proved to be more suitable for the present analysis. Although some authors choose to verify agreement through correlation methods, only reliability analysis takes into account the degree of agreement that could occur due to chance^(34)^. Koo and Li (2016) claim that, traditionally, paired *t* test and Bland-Altman plot are often used to assess reliability^(35)^. However, none of them constitute a measure of agreement analysis, since Pearson’s coefficient is only a correlation measure, not ideal for reliability measures. It is desirable that a reliability measure includes both the correlation measure and the agreement between the measures, which is achieved through ICC, a widely used method to calculate inter-rater, intra-rater and test-retest reliability. Such assessments are considered fundamental to assess how much the measures offered by a given assessment instrument are replicable and, therefore, reliable.

ICC values range from 0 to 1; values closer to 1 represent stronger reliability. It is suggested that ICC values less than 0.5 indicate low reliability; values between 0.5 and 0.75 indicate moderate reliability; values between 0.75 and 0.90 indicate good reliability; and values above 0.90 indicate excellent reliability. There are several ways to calculate ICC. In the present study, test-retest reliability used a mixed two-factor model (Two-Way Mixed-Effects Model), with absolute agreement.

Studies suggest the correlation between resilience and different social, economic, cultural, clinical and psychological aspects^(14-17,36)^. As for the relationship between resilience and cognitive functioning, Tomaszeuski *et al*. showed that the use of compensatory strategies can contribute to greater resilience among older adults, even in the context of a cognitive decline^(37)^. Fortes *et al*. (2009)^(10)^ demonstrated a positive and significant correlation between resilience and cognitive performance. Likewise, another study, with military veterans aged 52 to 101 years, demonstrated that higher psychological resilience was associated with better cognitive performance^(38)^.

The assessment protocol of the present study did not include all variables potentially correlated with resilient behavior. For instance, there is a discussion as to whether resilience is a state or a trait. Chen *et al*. (2017) investigated whether personality traits - neuroticism and conscientiousness - are moderating factors in the relationship between perceived stress and depressive symptoms in Chinese older adults living in the United States. The results suggested a moderating effect of conscientiousness, as the positive relationship between perceived stress and depressive symptoms was weaker in individuals with higher levels of conscientiousness^(39)^.

In a study, Sheerin *et al*. (2018) assessed the mitigating effect of resilience on the development of major depression and generalized anxiety disorders. The results showed that the relationship between resilience and the impact of stressful events throughout life was significant. Even in the context of a large number of stressful events, resilience had a protective effect against the development of mental disorders such as depression and anxiety related conditions^(40)^.

Although there has been a greater attention to the aging population, most related initiatives and research still remain focused on high-income countries, even though demographic studies estimate that, in 2050, more than two-thirds of older adults will be living in less developed countries. Moreover, the fastest increase in older adults is expected to happen in least developed countries from 2019 to 2050^(41-42)^. Besides, older people from developing countries may face even more difficulties than those from developed countries^(43)^.

### Study limitations

It can be considered that the number of individuals assessed in retest, for reliability analysis, was also below average when compared to other validity studies. Inter-rater reliability assessment methodology, in addition to include few participants, was carried out through application in pairs. Despite being a valid method, this methodology increases the chance that the correlation coefficient is close to 1. Additionally, sample selection bias may have weakened the correlation between resilience and sociodemographic and epidemiological variables as well as the possibility of generalizing psychometric results.

### Contributions to nursing and health

Considering populational aging and the importance that resilience has for older adults, instruments to assess this parameter are needed. This study demonstrated that EMRII-BR is a valid and reliable instrument for measuring resilience in Brazilian older adults. Therefore, health professionals may implement this scale on clinical practice as well as EMRII-BR can be used in clinical research.

## CONCLUSION

The MIIRM went through a well-designed process of translation and cultural adaptation, resulting in the final version of the EMRII-BR.

The EMRII-BR validity study demonstrated that this instrument is valid for assessing resilience in older adults, with adequate internal consistency (α = 0.705). The choice of EFA was based on the assumption that the changes made in the process of translation and cultural validity as well as the results of the application in the target population justify the need to verify a new factor structure, potentially simpler than the one of the original study. Despite that, it would be interesting to conduct a confirmatory factor analysis, both for the structure presented in the original study and for the structure of the present study.

Finally, it is considered that these results offer new means of understanding aging positive factors, which is one of the most complex challenges in contemporary science. Therefore, access to duly adapted and validated instruments for each culture and population allows a more accurate assessment of aging in its particularities. It is essential that, based on such instruments, new research can investigate resilience in specific populations, such as older adults with mild cognitive impairment, older adults with a diagnosis of mood disorders, older adults with personality disorders, among others.

## Data Availability

https://doi.org/10.48331/scielodata.AXNCDN
